# Immune‐Array Analysis in Sporadic Inclusion Body Myositis Reveals HLA–DRB1 Amino Acid Heterogeneity Across the Myositis Spectrum

**DOI:** 10.1002/art.40045

**Published:** 2017-04-04

**Authors:** Simon Rothwell, Robert G. Cooper, Ingrid E. Lundberg, Peter K. Gregersen, Michael G. Hanna, Pedro M. Machado, Megan K. Herbert, Ger J. M. Pruijn, James B. Lilleker, Mark Roberts, John Bowes, Michael F. Seldin, Jiri Vencovsky, Katalin Danko, Vidya Limaye, Albert Selva‐O'Callaghan, Hazel Platt, Øyvind Molberg, Olivier Benveniste, Timothy R. D. J. Radstake, Andrea Doria, Jan De Bleecker, Boel De Paepe, Christian Gieger, Thomas Meitinger, Juliane Winkelmann, Christopher I. Amos, William E. Ollier, Leonid Padyukov, Annette T. Lee, Janine A. Lamb, Hector Chinoy, for the Myositis Genetics Consortium, Christopher Denton, Karina Gheorghe, David Hilton‐Jones, Patrick Kiely, Herman Mann

**Affiliations:** ^1^ University of Manchester Manchester UK; ^2^ University of Liverpool Liverpool UK; ^3^ Karolinska University Hospital, Karolinska Institutet Stockholm Sweden; ^4^ Feinstein Institute for Medical Research Manhasset New York; ^5^ University College London London UK; ^6^ Beth Israel Deaconess Medical Center, Boston, Massachusetts, and Radboud University Nijmegen Nijmegen The Netherlands; ^7^ Radboud University Nijmegen Nijmegen The Netherlands; ^8^ University of Manchester, Manchester, UK, and Salford Royal NHS Foundation Trust Salford UK; ^9^ Salford Royal NHS Foundation Trust Salford UK; ^10^ University of California Davis; ^11^ Charles University Prague Czech Republic; ^12^ University of Debrecen Debrecen Hungary; ^13^ Royal Adelaide Hospital, Adelaide South Australia Australia; ^14^ Vall d'Hebron General Hospital Barcelona Spain; ^15^ University of Oslo Oslo Norway; ^16^ Hôpital Pitié‐Salpêtrière, UPMC Paris France; ^17^ University Medical Center Utrecht Utrecht The Netherlands; ^18^ University of Padova Padua Italy; ^19^ Ghent University Hospital Ghent Belgium; ^20^ Helmholtz Zentrum München Neuherberg Germany; ^21^ Technische Universität München, Munich, Germany, and Helmholtz Zentrum München Neuherberg Germany; ^22^ Dartmouth College Hanover New Hampshire; ^23^ Karolinska Institutet Stockholm Sweden; ^24^ Central Manchester University Hospitals NHS Foundation Trust, University of Manchester Manchester UK

## Abstract

**Objective:**

Inclusion body myositis (IBM) is characterized by a combination of inflammatory and degenerative changes affecting muscle. While the primary cause of IBM is unknown, genetic factors may influence disease susceptibility. To determine genetic factors contributing to the etiology of IBM, we conducted the largest genetic association study of the disease to date, investigating immune‐related genes using the Immunochip.

**Methods:**

A total of 252 Caucasian patients with IBM were recruited from 11 countries through the Myositis Genetics Consortium and compared with 1,008 ethnically matched controls. Classic HLA alleles and amino acids were imputed using SNP2HLA.

**Results:**

The HLA region was confirmed as the most strongly associated region in IBM (*P* = 3.58 × 10^−33^). HLA imputation identified 3 independent associations (with HLA–DRB1*03:01, DRB1*01:01, and DRB1*13:01), although the strongest association was with amino acid positions 26 and 11 of the HLA–DRB1 molecule. No association with anti–cytosolic 5′‐nucleotidase 1A–positive status was found independent of HLA–DRB1*03:01. There was no association of HLA genotypes with age at onset of IBM. Three non‐HLA regions reached suggestive significance, including the chromosome 3 p21.31 region, an established risk locus for autoimmune disease, where a frameshift mutation in *CCR5* is thought to be the causal variant.

**Conclusion:**

This is the largest, most comprehensive genetic association study to date in IBM. The data confirm that HLA is the most strongly associated region and identifies novel amino acid associations that may explain the risk in this locus. These amino acid associations differentiate IBM from polymyositis and dermatomyositis and may determine properties of the peptide‐binding groove, allowing it to preferentially bind autoantigenic peptides. A novel suggestive association within the chromosome 3 p21.31 region suggests a role for *CCR5*.

Sporadic inclusion body myositis (IBM) is an acquired muscle disease characterized clinically by weakness and muscle wasting, predominantly of the quadriceps and long finger flexor muscles. While degenerative changes are recognized, there are also immune‐mediated mechanisms at play, characterized by inflammatory features in muscle biopsy specimens and the presence of circulating autoantibodies. These autoantibodies include anti‐Ro and a recently identified autoantibody directed against cytosolic 5′‐nucleotidase 1A (anti‐cN1A), which is present in approximately one‐third of patients 1, 2. While the primary cause of the disease remains unknown, genetic factors may influence disease susceptibility. A group of hereditary diseases that includes the hereditary inclusion body myopathies and other muscular dystrophies such as the myofibrillary myopathies may mimic clinical features of IBM 3. These diseases may also exhibit similar pathologic features, such as rimmed vacuoles and protein accumulations; clinical and histopathologic suspicion of these diseases should prompt appropriate genetic testing.

To date, the strongest genetic risk identified for IBM lies within the major histocompatibility complex (MHC), in particular with HLA–DRB1*03:01, an allele present on the 8.1 ancestral haplotype that is a risk factor for many autoimmune diseases, including the idiopathic inflammatory myopathies (IIMs) 4. Other HLA–DRB1 alleles such as HLA–DRB1*01:01 and HLA–DRB1*13:01 have also been implicated in IBM, and genotypic combinations of these alleles have been reported to correlate with clinical phenotype 5.

Candidate gene studies in IBM have focused mainly on the MHC, and there are few validated associations outside of this region. Genes associated with neurodegenerative diseases such as Alzheimer's Disease have been examined in IBM, for example, the genes for β/A4‐amyloid precursor protein and apolipoprotein E, although these studies frequently have shown negative or conflicting results 6, 7, 8. Other candidate gene approaches have focused on autoantibody targets 1 and genes previously implicated in hereditary inclusion body myopathies 9, 10. However, those studies also have failed to find significant common associations.

We recently reported a genetic association study in polymyositis (PM) and dermatomyositis (DM) using the Immunochip array, a custom‐designed, high‐density genotyping chip that covers genes known to be associated with a variety of autoimmune diseases 11. Samples from patients with IBM were genotyped concurrently and were analyzed separately in this analysis using a previously described method of case–control matching to control for population differences. Using the Immunochip, we have conducted the largest genetic study to date in IBM to investigate potential associations with immune‐related genes, and we have used imputation to refine associations within the MHC.

## PATIENTS AND METHODS

### Study populations

A total of 252 patients with IBM from 11 countries were recruited through the Myositis Genetics Consortium (MYOGEN). A list of MYOGEN study investigators in addition to the authors of this article is provided in Appendix A. Written informed consent was obtained from all patients with approval from research ethics boards at each participating center. Patients with IBM were included if they fulfilled the following criteria: Griggs (“definite” or “possible”) 12, Medical Research Council (“pathologically defined,” “clinically defined,” or “possible”) 13, or European Neuromuscular Centre (“clinico‐pathologically defined,” “clinically defined,” or “probable”) 14. Age at onset for UK patients with IBM was the age at onset of first symptoms as recorded in the clinical record.

Shared control samples from Sweden (the Epidemiological Investigation of Rheumatoid Arthritis study), Spain, and The Netherlands were provided by the Rheumatoid Arthritis Consortium International 15, with control samples from the UK provided by the Wellcome Trust Case Control Consortium 16. Control samples from Italy, Norway, Belgium, and France were provided by the International Multiple Sclerosis Genetics Consortium 17. Polish and Hungarian control samples were provided by the Celiac Consortium 18, and German control samples were provided by the KORAgen consortium 19.

### Genotyping and quality control

Genotyping was performed in accordance with Illumina's protocols in the UK (Centre for Genetics and Genomics Arthritis Research UK, University of Manchester, Manchester, UK) and the US (Feinstein Institute for Medical Research, Manhasset, NY). Standard quality control was performed as described previously 11. Four controls for each case were matched for ethnicity using principal components analysis coordinates using a method described previously 20.

### Statistical analysis

Statistical analyses were performed in Plink version 1.7 (http://zzz.bwh.harvard.edu/plink/index.shtml) using a logistic regression applying an additive model. Sex and population differences were controlled for by including sex and the top 10 principal components as covariates. Significance was defined as *P* < 5 × 10^−8^. We also reported variants reaching a second tier of significance of *P* < 2.25 × 10^−5^, calculated using the genetic Type I Error Calculator 21. Odds ratios (ORs) are provided with 95% confidence intervals (95% CIs).

To investigate associations with HLA and age at onset, linear regressions were used, with *P* values less than 0.05 considered significant. Analyses were carried out using Stata statistical software version 13.1 (StataCorp).

### Functional annotation

Evidence for functional effects and expression quantitative trait loci (eQTLs) were investigated for the lead single‐nucleotide polymorphisms (SNPs) in each region, and SNPs in high linkage disequilibrium (LD) (r^2^ ≥ 0.8) were obtained from Phase 3 1000 Genomes data using LDlink 22.

### MHC imputation and association analysis

Classic HLA alleles and corresponding amino acid sequences were imputed from Immunochip SNP data using the SNP2HLA program as described previously 11. Significance was defined as *P* < 6.8 × 10^−6^ based on a Bonferroni correction of the 7,323 markers imputed by SNP2HLA 23. For consistency, the most associated variant was used in the stepwise conditional analysis. Molecular graphics were generated and analyses were performed with the University of California, San Francisco (UCSF) Chimera package version 1.10.2 (Resource for Biocomputing, Visualization, and Informatics at UCSF).

### Anti‐cN1A detection

Enzyme‐linked immunosorbent assay detection of anti‐cN1A antibodies was performed using the optimized protocol as described previously 24. Briefly, biotinylated peptides were incubated on Streptawell High Bind microplates (Roche) for 1 hour at 37°C to immobilize the peptides. Unbound peptides were removed by washing the microplates 3 times. Diluted patient serum was then added to the microplate followed by incubation at 37°C for 1 hour. Unbound antigen was removed by further washing the microplate 5 times. Diluted rabbit anti‐human Ig was then added, and the plate was incubated for 1 hour at 37°C followed by a further 5 washes. Finally, the bound antibodies were visualized by adding substrate solution, and the reaction was stopped after 5 minutes by adding a stop solution. Signals were quantified by determining optical densities at 450 nm (OD_450 nm_). The OD_450 nm_ value corresponding to the highest Youden Index [calculated as: ([sensitivity/100] + [specificity/100] − 1)] 25 at which ≥98% specificity was achieved was chosen for each peptide. Sera were assessed as reactive if they were above the established cutoff value for at least one of the peptide antigens 24.

## RESULTS

### Genotyping quality control

After stringent SNP and sample quality control, we analyzed 104,636 genetic variants in 252 patients with IBM and 1,008 ethnically matched controls (Table 1). Including the top 10 principal components as covariates and calculating the genomic inflation on a set of null SNPs gave a λ_gc_ of 1.04, indicating that patients and controls were well matched for ethnicity (see Supplementary Figure 1, available on the *Arthritis & Rheumatology* web site at http://onlinelibrary.wiley.com/doi/10.1002/art.40045/abstract).

**Table 1:** Numbers of samples from patients with inclusion body myositis and ethnically matched controls included in the analysis after quality control, by country of origin^a^

	Patients	Controls
Australia	44	–
Belgium	6	23
Czech Republic	2	–
France	19	35
Germany	–	29
Hungary	2	7
Italy	2	44
The Netherlands	9	69
Norway	1	30
Poland	–	8
Sweden	31	97
Spain	8	28
UK	128	485
US	–	153
Total	252	1,008

a Control samples were shared from Immunochip consortia. Four controls for each patient were matched based on nearest neighbor by principal components analysis coordinates.

### HLA is the most strongly associated region in IBM

**Figure 1:**
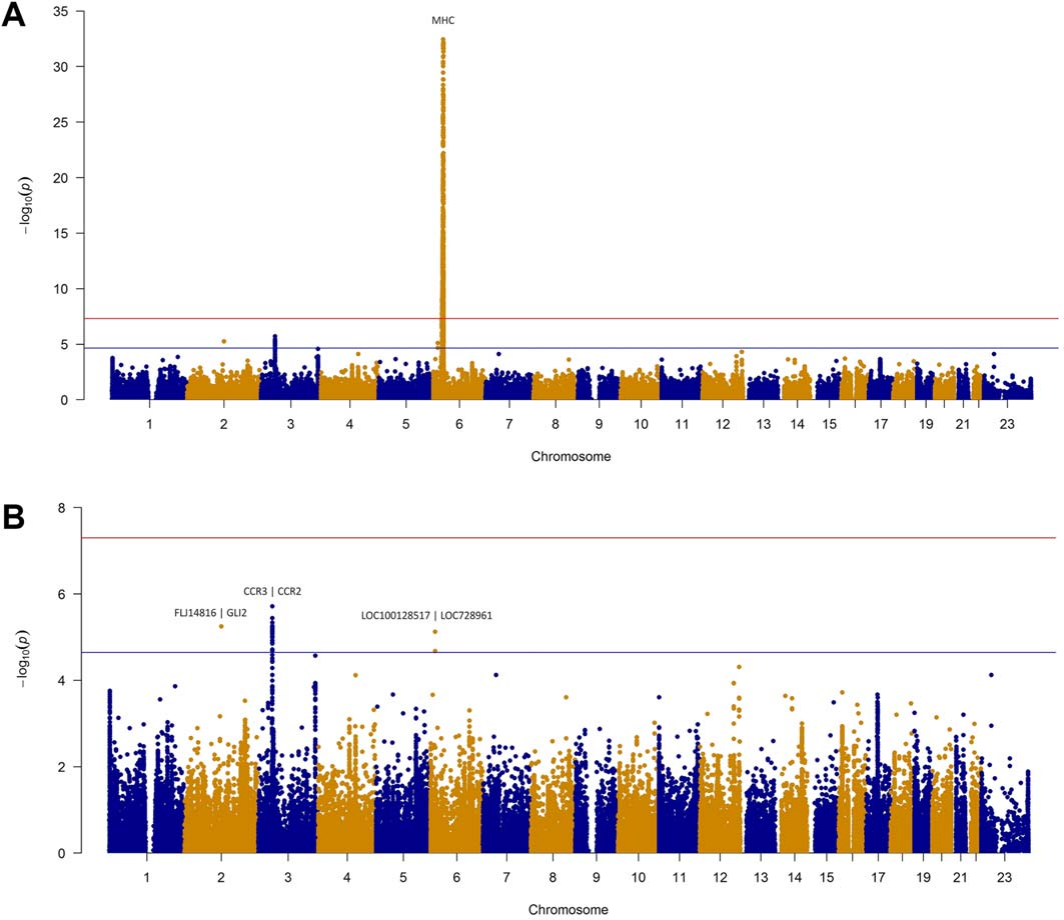
Manhattan plots of the inclusion body myositis (IBM) analysis. Red line represents genome‐wide level of significance (*P* < 5 × 10^−8^); blue line represents suggestive significance (*P* < 2.25 × 10^−5^). Shown is the analysis of 252 patients with IBM and 1,008 matched controls. **A,** Manhattan plot of the total Immunochip analysis. **B,** Manhattan plot of the IBM analysis with the major histocompatibility complex (MHC) region (chromosome 6 25–35) removed for visualization purposes. Color figure can be viewed in the online issue, which is available at http://onlinelibrary.wiley.com/journal/doi/10.1002/art.40045/abstract.

SNPs within the MHC region were the only variants reaching genome‐wide significance of *P* < 5 × 10^−8^ (Figure 1A). The strongest association was with rs3129950 (*P* = 3.58 × 10^−33^), a SNP intronic of *LOC101929163* and 3´ of *BTNL2* (Table 2). As the Immunochip contains high‐density SNP coverage across the MHC, this region was subsequently analyzed separately using HLA imputation in an attempt to refine this association to a functional gene. Initially, genes independent of the MHC were investigated that reached a suggestive level of significance.

**Table 2:** Analysis of the 252 patients with inclusion body myositis compared to 1,008 ethnically matched controls^a^

Gene region	Chr.	Position	SNP	Minor allele	MAF in patients	MAF in controls	*P*	OR (95% CI)	Localization of LD to nearest genes (r^2^ ≥ 0.9)
*MHC*	6	32358201	rs3129950	C	0.34	0.11	3.58 × 10^−33^ b	5.69 (4.28–7.55)	*MHC*
*CCR3/CCR2*	3	46389462	rs112088397	T	0.08	0.16	1.93 × 10^−6^ c	0.42 (0.29–0.60)	Downstream of *CCR3* to intron 2 of *LTF*; incorporating *CCR2*, *CCR5*, and *CCRL2*
*FLJ14816/GLI2*	2	121338584	rs1880542	T	0.56	0.45	5.66 × 10^−6^ c	1.60 (1.31–1.96)	Intergenic of *FLJ14816* and *GLI2*
*LOC100128517/LOC728961*	6	14560180	rs9396510	T	0.11	0.05	7.52 × 10^−6^ c	2.23 (1.57–3.17)	Intergenic of *LOC100128517* and *LOC728961*

a Coordinates are based on the human assembly GRCh37. Chr. = chromosome; SNP = single‐nucleotide polymorphism; MAF = minor allele frequency; OR = odds ratio; 95% CI = 95% confidence interval; LD = linkage disequilibrium.

b Reported at genome‐wide significance (*P* < 5 × 10^−8^).

c Reported at second tier of significance (*P* < 2.25 × 10^−5^).

### Suggestive significance of 3 non‐HLA associations

**Figure 2:**
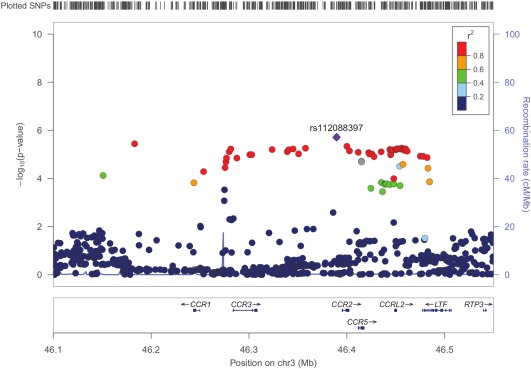
Regional association plot of the chromosome 3 (Chr3) p21.31 region in inclusion body myositis. The plot shows strength of association (−log_10_[*P*]) against chromosomal position. The most strongly associated single‐nucleotide polymorphism (SNP) is colored purple, with other SNPs colored by the degree of linkage disequilibrium (r^2^). Local recombination rates estimated from the HapMap population of Utah residents with ancestry from northern and western Europe are plotted against the secondary y‐axis, showing recombination hotspots across the region.

Three non‐HLA regions reached our second tier of significance (defined as *P* < 2.25 × 10^−5^) calculated using the genetic Type I Error Calculator 21. Figure 1B shows the Manhattan plot with the MHC region removed for visualization purposes. The most convincing associations were in the chromosome 3 p21.31 region, where 49 SNPs reached the suggestive level of significance (Figure 2). The strongest association was upstream of *CCR2* (rs112088397, *P* = 1.93 × 10^−6^, OR 0.42 [95% CI 0.29–0.60]); however, this haplotype contains many genes including *CCR3*, *CCR2*, *CCR5*, and *CCRL2*.

Potentially functional variants were investigated for all non‐HLA SNPs reaching suggestive significance and those tagged by the lead SNP (r^2^ ≥ 0.8) (see Supplementary Tables 1–3, http://onlinelibrary.wiley.com/doi/10.1002/art.40045/abstract). Multiple SNPs within the chromosome 3 p21.31 region had evidence for eQTLs for the expression of *CCR5* in monocytes 26. There was 1 missense SNP in the chromosome 3 p21.31 region (rs6441977) that was predicted to be “benign” by PolyPhen‐2 27; however, a frameshift mutation (rs333) is a known variant that results in a 32‐bp deletion and a nonfunctional receptor. Conditional analysis on this locus did not identify additional independent variants.

### HLA imputation reveals association with HLA–DRB1

To refine associations within the MHC region, HLA alleles were imputed from SNP genotyping information using SNP2HLA 23. Variants reaching statistical significance (*P* < 6.8 × 10^−6^) after each round of conditioning are included in Supplementary Tables 4–6, http://onlinelibrary.wiley.com/doi/10.1002/art.40045/abstract. The strongest associations were with alleles that are part of the 8.1 ancestral haplotype, with HLA–DRB1*03:01 reaching *P* = 5.77 × 10^−34^ (OR 7.97 [95% CI 5.88–10.95]). Due to strong LD within the MHC, other alleles within this haplotype such as HLA–DQB1*02:02 and HLA–B*08:01 also had strong associations in this analysis; however, these lost significance after conditioning on the effect of HLA–DRB1*03:01. Conducting stepwise conditional analysis on significant HLA–DRB1 alleles, an independent effect was seen with HLA–DRB1*01:01 (*P* = 1.56 × 10^−16^, OR 4.64 [95% CI 3.33–6.49]). A further independent effect was seen with HLA–DRB1*13:01 (*P* = 3.28 × 10^−8^, OR 3.19 [95% CI 2.14–4.72]).

As risk may lie within multiple HLA alleles, we imputed amino acids to investigate whether shared positions within risk alleles might explain the risk at this locus (see Supplementary Tables 7 and 8, http://onlinelibrary.wiley.com/doi/10.1002/art.40045/abstract). Amino acid position 26 of HLA–DRB1 was associated more significantly than a single classic HLA allele (*P* = 5.22 × 10^−43^), with risk attributable to a tyrosine at this position (OR 3.83 [95% CI 2.80–5.29]) (Table 3). This contrasts with PM and DM, where HLA–DRB1 amino acid position 77 was the most strongly associated position (Table 3). Conditioning on the effects of position 26 in IBM revealed an independent effect of position 11 of HLA–DRB1 (*P* = 3.80 × 10^−13^). At this position, serine is the most common amino acid in the population and was therefore used as the reference. As many other amino acids are protective at this position, we can infer that serine confers the greatest risk. No further amino acid positions were statistically significant after conditioning on positions 26 and 11 of HLA–DRB1.

**Table 3:** Independent associations of HLA–DRB1 amino acids in clinical subgroups of idiopathic inflammatory myopathy^a^

Association, marker	*P*	OR (95% CI)
Most associated in inclusion body myositis		
Position 26	Omnibus 5.22 × 10^−43^	
Phenylalanine	Reference^b^	1
Tyrosine	1.19 × 10^−16^	3.83 (2.8–5.29)
Leucine	0.32	0.63 (0.24–1.48)
Position 11	Omnibus 3.80 × 10^−13^	
Serine	Reference^b^	1
Proline	9.06 × 10^−5^	0.42 (0.27–0.64)
Valine	2.25 × 10^−6^	0.33 (0.2–0.51)
Glycine	4.46 × 10^−6^	0.26 (0.14–0.45)
Leucine	0.03	2.75 (1.15–7.5)
Aspartic acid	1.41 × 10^−3^	0.13 (0.03–0.39)
Most associated in polymyositis^c^		
Position 77	Omnibus 1.65 × 10^−80^	
Threonine	Reference^b^	1
Asparagine	1.65 × 10^−80^	2.93 (2.53–3.17)
Most associated in dermatomyositis^c^		
Position 77	Omnibus 1.37 × 10^−36^	
Threonine	Reference^b^	1
Asparagine	1.37 × 10^−36^	2.14 (1.90–2.41)

a *P* values and odds ratios (ORs) with 95% confidence intervals (95% CIs) were calculated in a logistic regression.

b Reference amino acid is taken as the most frequent in the population.

c For comparative purposes, HLA–DRB1 amino acid association statistics for polymyositis and dermatomyositis are shown (from ref. 11).

### No distinct HLA association with anti‐cN1A positivity

Anti‐cN1A antibodies were detected in 36 of the 104 patients serologically tested (35%). After quality control, HLA imputation was conducted on 35 anti‐cN1A–positive patients and 140 healthy controls, and the most significant 4‐digit classic HLA association was found with HLA–DRB1*03:01 (*P* = 5.62 × 10^−8^, OR 11.52 [95% CI 4.95–29.29]) (see Supplementary Table 9, http://onlinelibrary.wiley.com/doi/10.1002/art.40045/abstract). After stepwise conditional analysis, we did not find any additional independent effects. We then compared the 35 anti‐cN1A–positive patients with 68 anti‐cN1A–negative patients, and we found no significant differences in HLA associations between these groups (data not shown).

### No effect of HLA–DRB1 allele interactions on age at onset

Previous studies have suggested that HLA–DRB1 alleles may have disease‐modifying effects in IBM, with the HLA–DRB1*03/*01 genotype conferring an earlier age at onset and more severe muscle weakness 5, 28. HLA and age at onset data were available for 124 UK patients with IBM. Linear regression was used to analyze the relationship of HLA–DRB1 alleles with age at onset. No significant associations were found with risk alleles HLA–DRB1*01:01, DRB1*03:01, or DRB1*13:01 when these were analyzed separately or in combination (see Supplementary Table 10, http://onlinelibrary.wiley.com/doi/10.1002/art.40045/abstract).

## DISCUSSION

This is the largest genetic association study to date in Caucasian patients with IBM. The results confirm that HLA is the most strongly associated region, identify multiple HLA–DRB1 alleles conferring risk, and suggest amino acid positions that may explain the risk in this locus. A novel suggestive association within the chromosome 3 p21.31 locus indicates genetic overlap with other autoimmune diseases and identifies a potentially functional variant that may contribute to the pathogenesis of IBM.

HLA imputation confirmed that the strongest risk within this region lies with HLA–DRB1*03:01. Stepwise conditional analyses revealed additional independent associations with HLA–DRB1*01:01 and HLA–DRB1*13:01, suggesting that the HLA–DRB1 gene is important in susceptibility to IBM. In contrast to previous studies, there were no significant associations of age at onset with HLA–DRB1 alleles. Other reported disease‐modifying effects of HLA alleles in IBM, such as with disease severity and lower quadriceps muscle strength, were not investigated due to a lack of consistent clinical data across this multinational, multicenter study. Previous studies have also investigated additional risk factors present on the MHC, such as polymorphisms in the gene for Notch‐4 29 or carriage of secondary HLA–DRB loci such as HLA–DRB3 30. Although not explicitly investigated in the present study, these associations are in strong linkage with the 8.1 ancestral haplotype, and we do not expect our data to differentiate between these risk factors. Conditioning on the presence of HLA–DRB1, no additional genetic variants within the MHC region were associated with IBM. The frequency of genotypes among patients with IBM (see Supplementary Table 10, http://onlinelibrary.wiley.com/doi/10.1002/art.40045/abstract) suggests that patients homozygous for HLA–DRB1*03:01 and DRB1*01:01 are at lower risk of disease. The contribution of nonadditive effects across HLA alleles has been reported in several autoimmune diseases and may explain higher risk for heterozygote individuals 31. The small numbers in this cohort mean that the study is underpowered to statistically test this in IBM.

**Figure 3:**
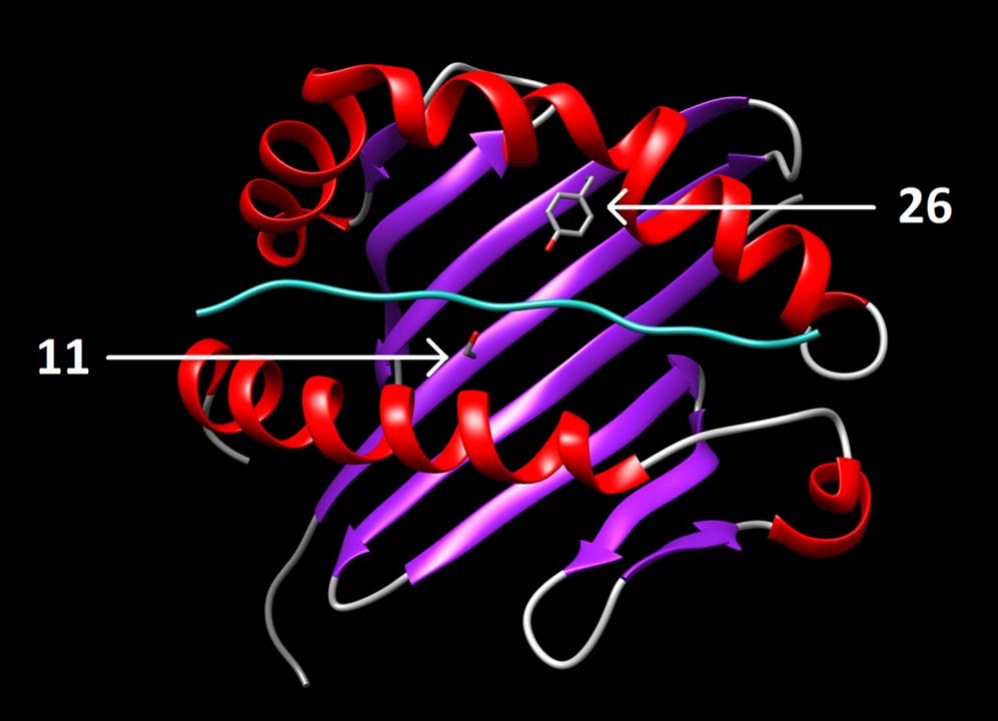
Locations of positions 26 and 11 of HLA–DRB1 within DR β‐chain 1. Positions 26 and 11 are independently associated with inclusion body myositis. **Arrows** indicate the locations of the risk‐conferring amino acids Tyr^26^ and Ser^11^ within the β‐sheet floor of DR β‐chain 1. Color figure can be viewed in the online issue, which is available at http://onlinelibrary.wiley.com/journal/doi/10.1002/art.40045/abstract.

As multiple HLA–DRB1 alleles were associated with IBM, we investigated whether there were shared amino acid positions within HLA–DRB1 risk alleles that might explain the risk at this locus. Position 26 of HLA–DRB1 was more strongly associated than a classic HLA allele alone (*P* = 5.22 × 10^−43^ versus *P* = 5.77 × 10^−34^). An additional independent effect was found with position 11 of HLA–DRB1. Positions 11 and 26 have been associated previously with seropositive autoimmune disease, such as systemic lupus erythematosus 32, which suggests that these positions may determine properties of the HLA–DRB1 peptide‐binding groove, allowing it to preferentially bind autoantigenic peptides (Figure 3). Certain amino acids, such as a tyrosine at position 26 and serine at position 11, were associated with risk in this analysis; however, a lack of statistical power means that we were unable to completely characterize the effects of certain amino acids in this molecule. It is interesting to note that while IBM shares HLA–DRB1*03:01 as a significant risk factor with other inflammatory myopathies, such as PM and DM, the amino acid associations differ between these subtypes. In PM and DM, amino acid position 74 of HLA–DRB1 explains almost all of the risk within this gene 11. These differences in amino acid associations may be explained by the additional independent effects of HLA–DRB1*01:01 and HLA–DRB1*13:01 in IBM that are not associated with PM or DM. Understanding the peptide‐binding specificities of these risk alleles may inform future research along with the potential identification of unique autoantigens presented to the immune system in IBM.

We investigated potential associations between specific HLA alleles and the newly described anti‐cN1A antibody. The significant association observed with HLA–DRB1*03:01 and the anti‐cN1A antibody may be due to an increased association with HLA–DRB1*03:01 as a whole in IBM. No significant differences in HLA associations were observed between anti‐cN1A–positive and anti‐cN1A–negative patients. A recent study in an Australian cohort of patients also failed to show any association with anti‐cN1A antibodies and MHC class II alleles other than HLA–DR3 33. Furthermore, we do not have complete data on co‐occurrence of anti‐cN1A and other antibodies, such as anti‐Ro, which also has a strong HLA–DR3 association 34. It may be that a significantly larger sample size is needed to detect novel HLA associations in patients with anti‐cN1A antibodies.

The Immunochip is a custom‐designed chip that contains a dense set of SNPs covering 186 loci based on evidence of association with 12 different autoimmune and inflammatory diseases 18. Therefore, the current study tests a specific hypothesis that IBM shares genetic overlap with other autoimmune diseases. The current study has not comprehensively tested other loci that have been purported to be associated with IBM, such as those predisposing to hereditary inclusion body myopathies or loci associated with other degenerative diseases. The observation that the MHC region is strongly associated, and evidence of association with other genes on this array, suggests an immune‐mediated component to IBM. It is not clear whether this represents a primary or secondary involvement.

No non‐HLA loci investigated reached genome‐wide significance, although this is to be expected in a study of this size. While comparatively large for IBM, this study is underpowered to detect associations of small effect sizes that are expected in genetic studies of conditions with complex etiologies. Three loci did reach a suggestive level of significance (*P* < 2.25 × 10^−5^). Of particular interest is the chromosome 3 p21.31 region, which is known to be associated with multiple autoimmune diseases such as celiac disease, type 1 diabetes mellitus, and Behçet's disease and is suggestively associated in juvenile idiopathic arthritis (JIA) 35, 36, 37, 38. The strongest association in this region was with rs112088397, which tags a large haplotype block where many additional SNPs reached a suggestive level of significance and is the same risk haplotype as that reported in JIA (r^2^ = 0.87) 35, 39. The variant rs112088397 in our study is found at a higher frequency in controls (minor allele frequency [MAF] of 0.08 for patients versus MAF of 0.16 for controls) and is therefore protective against IBM (OR 0.42 [95% CI 0.29–0.60]). Proxies for rs112088397 fall within multiple candidate genes including *CCR1*, *CCR3*, *CCR2*, *CCR5*, and *CCRL2*, and therefore it is difficult to identify the causal variant in this region. Interestingly, this haplotype contains a frameshift mutation (rs333) that results in a 32‐bp deletion variant (CCR5Δ32) and a nonfunctional receptor. The most strongly associated non‐HLA SNP in IBM is in high LD with this frameshift mutation (r^2^ = 0.86); furthermore, a number of SNPs in this region are eQTLs for the expression of *CCR5* in monocytes 26.

CCR5 binds a number of proinflammatory chemokines that are up‐regulated in IIMs and IBM, such as CCL3 (macrophage inflammatory protein 1α [MIP‐1α]), CCL4 (MIP‐1β), and CCL5 (RANTES). CCR5 has been shown to be predominantly expressed on monocytes, macrophages, and T cells, up‐regulated in IBM muscle tissue, and localized on inflammatory cells invading nonnecrotic muscle fibers 40, 41. Interestingly, in rheumatoid arthritis (RA) the density of CCR5 molecules on the T cell surface determines efficiency of its function as a chemokine receptor and intensity of T cell migration toward RA synoviocytes 42. We hypothesize that CCR5 is important in the pathogenesis of IBM, consistent with studies showing an up‐regulation of CCR5 in muscle tissue of patients. Individuals with the protective rs333 frameshift mutation described above will carry a nonfunctional variant and/or decreased expression of CCR5, resulting in reduced migration of T cells into muscle fiber.

It is interesting that the suggestive association with the chromosome 3 p21.31 region in this study was found with only 252 individuals. This may be explained by the stronger effect size in IBM compared to JIA (0.42 versus 0.78, respectively) 35, and although IBM is a rare disease, it may mean that replication of this association is possible with ongoing sample collection. Due to the rarity of IBM, it is difficult to ascertain the sample sizes needed for genome‐wide association studies; therefore, next‐generation sequencing could be an approach to detect rare, potentially functional variants of large effect size. Sequencing studies are currently underway taking either a candidate gene approach 10 or a hypothesis‐free approach sequencing exomes of a large number of patients with IBM 9. The present study has not comprehensively tested other loci that have been purported to be associated with IBM, such as those predisposing to hereditary inclusion body myopathies or loci associated with other degenerative diseases. In a disease in which the etiology is unknown, sequencing could be successful in identifying novel variants and/or pathways involved in disease pathogenesis.

In summary, we have conducted the largest genetic association study to date in Caucasian patients with IBM, confirming the involvement of an immune‐mediated genetic component of this understudied disease. Studies in the genetics of IBM are hampered by small sample sizes due to the rarity of this disease. Ongoing sample collection, as well as further international collaborative studies, will allow us to further characterize genetic influences on susceptibility to IBM.

## AUTHOR CONTRIBUTIONS

All authors were involved in drafting the article or revising it critically for important intellectual content, and all authors approved the final version to be published. Dr. Rothwell had full access to all of the data in the study and takes responsibility for the integrity of the data and the accuracy of the data analysis.

### Study conception and design

Rothwell, Cooper, Lundberg, Gregersen, Ollier, Lamb, Chinoy.

### Acquisition of data

Rothwell, Cooper, Lundberg, Gregersen, Hanna, Machado, Herbert, Pruijn, Roberts, Vencovsky, Danko, Limaye, Selva‐O'Callaghan, Platt, Molberg, Benveniste, Radstake, Doria, De Bleecker, De Paepe, Gieger, Meitinger, Winkelmann, Padyukov, Lee, Lamb, Chinoy.

### Analysis and interpretation of data

Rothwell, Cooper, Lundberg, Lilleker, Bowes, Seldin, Amos, Ollier, Padyukov, Lamb, Chinoy.

## Supporting information

Supplementary Materials 1

Supplementary Materials 2

## APPENDIX A MYOGEN INVESTIGATORS

Study investigators of the Myositis Genetics Consortium, in addition to the authors of this article, are as follows: Drs. Christopher Denton (Royal Free Hospital, London, UK), Karina Gheorghe (Karolinska Institutet, Stockholm, Sweden), David Hilton‐Jones (John Radcliffe Hospital, Oxford, UK), Patrick Kiely (St. George's Hospital, London, UK), and Herman Mann (Institute of Rheumatology, Prague, Czech Republic).
