# Supplementary material for: Immune‐Array Analysis in Sporadic Inclusion Body Myositis Reveals HLA–DRB1 Amino Acid Heterogeneity Across the Myositis Spectrum
Source: Arthritis Rheumatol. 2017 Apr 4;69(5):1090–9. doi: 10.1002/art.40045 (PMC5516174; doi:10.1002/art.40045)
Supplement: Supplementary file 1 — Supplementary Materials 1 [file ART-69-1090-s001.pdf]

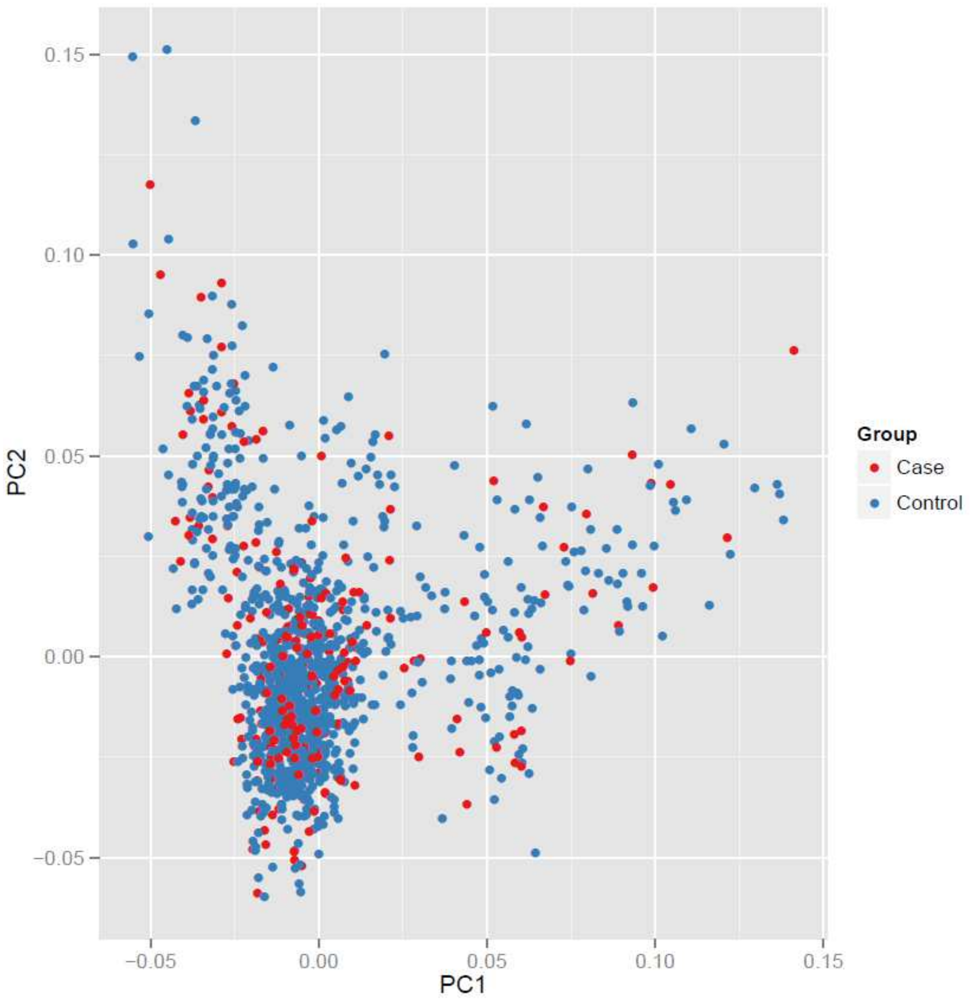

Supplementary Figure 1 - First two principal components of 252 IBM cases and 1,008 ethnically matched controls.

First two principal components of 252 inclusion body myositis cases and 1,008 ethnically matched controls. λ=1.04 indicates cases and controls are well matched for ethnicity.

RS Number	Coordinates	r^2	RegulomeDB	Function	Immunochip P-Value
rs112088397	chr3:46389462	Index	5	NA	1.93E-06
rs58697594	chr3:46275570	1	4	NA	3.52E-05
rs73833032	chr3:46276490	1	6	NA	2.06E-05
rs73833033	chr3:46277002	1	6	NA	1.41E-05
rs74433128	chr3:46279933	1	6	NA	7.66E-06
rs76733709	chr3:46281461	1	.	NA	5.97E-06
rs113676185	chr3:46286046	1	.	NA	.
rs60020651	chr3:46287651	1	.	NA	1.41E-05
rs146309725	chr3:46290767	1	.	NA	.
rs145617407	chr3:46293070	1	5	NA	.
rs75994669	chr3:46300863	1	.	NA	1.02E-05
rs79539493	chr3:46302938	1	5	NA	1.02E-05
rs138814707	chr3:46317354	1	6	NA	.
rs78372062	chr3:46323679	1	.	NA	6.23E-06
rs78729754	chr3:46339185	1	2b	NA	7.66E-06
rs80054040	chr3:46340285	1	5	NA	.
rs79629761	chr3:46340630	1	5	NA	6.39E-06
rs75352297	chr3:46345151	1	.	NA	5.92E-06
rs368945717	chr3:46345341	1	.	NA	.
rs57093591	chr3:46350716	1	.	NA	9.44E-06
rs113507038	chr3:46357822	1	5	NA	5.53E-06
rs112384491	chr3:46360359	1	.	NA	.
rs113647051	chr3:46366857	1	6	NA	.
rs3092960	chr3:46400062	1	2b	synonymous	4.60E-06
rs762790	chr3:46402688	1	4	NA	7.14E-06
rs3136535	chr3:46409113	1	6	NA	.
rs41490645	chr3:46410137	1	4	NA	.
rs2856758	chr3:46411661	1	4	NA	8.15E-06
rs11575821	chr3:46422355	1	1f	NA	8.45E-06
rs7652037	chr3:46423063	1	6	NA	9.57E-06
rs34291293	chr3:46425647	1	3a	NA	9.57E-06
rs34445878	chr3:46425711	1	3a	NA	.
rs113263161	chr3:46425718	1	2b	NA	.
rs34971514	chr3:46427354	1	.	NA	.
rs111669359	chr3:46427616	1	4	NA	.
rs6787972	chr3:46427835	1	4	NA	1.23E-05
rs11298823	chr3:46428089	1	4	NA	.
rs112359020	chr3:46429391	1	5	NA	.
rs35388950	chr3:46429679	1	.	NA	.
rs75630441	chr3:46429717	1	6	NA	.
rs113634435	chr3:46431695	1	6	NA	.
rs111713092	chr3:46431751	1	5	NA	.
rs113934302	chr3:46432856	1	.	NA	.
rs111961837	chr3:46433146	1	.	NA	.
rs112982903	chr3:46433247	1	5	NA	.
rs112368251	chr3:46433967	1	5	NA	.
rs113229741	chr3:46433987	1	5	NA	.
rs113255842	chr3:46435161	1	3a	NA	7.80E-06
rs6798291	chr3:46443931	1	6	NA	6.24E-06

rs916093	chr3:46444794	1	6	NA	1.03E-05
rs916094	chr3:46444835	1	6	NA	9.34E-06
rs112903945	chr3:46445343	1	6	NA	6.45E-06
rs11574428	chr3:46446721	1	1f	NA	6.45E-06
rs11574429	chr3:46446810	1	4	NA	6.45E-06
rs11574434	chr3:46447915	1	3a	NA	.
rs6808835	chr3:46449864	1	5	synonymous	.
rs6441977	chr3:46450072	1	5	missense	6.45E-06
rs1140865	chr3:46450884	1	.	NA	5.87E-06
rs2157061	chr3:46451502	1	5	NA	5.87E-06
rs2157062	chr3:46451647	1	1f	NA	.
rs6762266	chr3:46452863	1	1f	NA	5.87E-06
rs6770674	chr3:46452873	1	5	NA	.
rs112384806	chr3:46453487	1	5	NA	.
rs113774564	chr3:46454682	1	5	NA	5.87E-06
rs113069082	chr3:46454688	1	5	NA	.
rs375524839	chr3:46454910	1	.	NA	.
rs111514151	chr3:46455754	1	5	NA	5.47E-06
rs66928113	chr3:46455996	1	.	NA	.
rs113942981	chr3:46457329	1	5	NA	6.23E-06
rs113490452	chr3:46458228	1	4	NA	.
rs6782522	chr3:46458505	1	5	NA	.
rs6791599	chr3:46458942	1	1f	NA	5.87E-06
rs111959715	chr3:46459050	1	6	NA	5.87E-06
rs6791789	chr3:46459121	1	.	NA	6.14E-06
rs113365357	chr3:46459291	1	4	NA	6.86E-06
rs112870257	chr3:46459398	1	5	NA	.
rs113888916	chr3:46459411	1	5	NA	.
rs113710186	chr3:46459914	1	4	NA	5.87E-06
rs111958022	chr3:46459956	1	4	NA	.
rs113064717	chr3:46460013	1	4	NA	5.87E-06
rs111410234	chr3:46460675	1	.	NA	.
rs112712359	chr3:46461783	1	6	NA	7.27E-06
rs79815064	chr3:46277577	0.95	.	NA	.
rs11574435	chr3:46447972	0.95	4	NA	1.01E-04
rs75719572	chr3:46183180	0.91	5	NA	3.61E-06
rs112720180	chr3:46193899	0.91	.	NA	.
rs112338830	chr3:46193902	0.91	.	NA	.
rs4452362	chr3:46470654	0.91	5	NA	.
rs112479910	chr3:46471119	0.91	5	NA	.
rs113595266	chr3:46471525	0.91	3a	NA	.
rs4453882	chr3:46472651	0.91	.	NA	.
rs7645306	chr3:46474670	0.91	4	NA	.
rs77648833	chr3:46474955	0.91	4	NA	.
rs75820382	chr3:46475122	0.91	5	NA	1.18E-05
rs56063910	chr3:46477826	0.91	6	NA	1.20E-05
rs75094754	chr3:46477830	0.91	6	NA	1.20E-05
rs111960587	chr3:46482049	0.91	5	NA	1.35E-05
rs368124274	chr3:46252708	0.87	6	NA	.
rs79893749	chr3:46253650	0.87	.	NA	5.16E-05
rs112160248	chr3:46260225	0.87	.	NA	.
rs113341849	chr3:46384204	0.86	.	NA	.

rs333	chr3:46414943	0.86	4	frameshift	2.03E-05
rs113010081	chr3:46457412	0.86	5	NA	2.56E-05
rs3176953	chr3:46243718	0.83	5	NA	1.50E-04

Supplementary Table 1 – All SNPs within $r^2 \geq 0.8$ of most associated SNP in the *CCR3 / CCR2* region

Coordinates based on GRCh37. r^2 calculated from CEU samples from Phase 3 of the 1000 Genomes Project. RegulomeDB key below.

RegulomeDB Key

1a - eQTL + TF binding + matched TF motif + matched DNase Footprint + DNase peak
1b - eQTL + TF binding + any motif + DNase Footprint + DNase peak
1c - eQTL + TF binding + matched TF motif + DNase peak
1d - eQTL + TF binding + any motif + DNase peak
1e - eQTL + TF binding + matched TF motif
1f - eQTL + TF binding / DNase peak
2a - TF binding + matched TF motif + matched DNase Footprint + DNase peak
2b - TF binding + any motif + DNase Footprint + DNase peak
2c - TF binding + matched TF motif + DNase peak
3a - TF binding + any motif + DNase peak
3b - TF binding + matched TF motif
4 - TF binding + DNase peak
5 - TF binding or DNase peak
6 - other

RS Number	Coordinates	r²	RegulomeDB	Function	Immunochip P-Value
rs1880542	chr2:121338584	Index	4	NA	5.66E-06
rs9308615	chr2:121338985	1	.	NA	.
rs2311600	chr2:121340676	1	5	NA	.
rs1869026	chr2:121341281	1	4	NA	.
rs11122802	chr2:121335866	0.9597	5	NA	.
rs12623454	chr2:121326297	0.9409	5	NA	.
rs4849906	chr2:121342814	0.704	5	NA	.
rs2871815	chr2:121345300	0.686	5	NA	.
rs11675851	chr2:121346579	0.686	5	NA	.
rs1109426	chr2:121345463	0.6685	2b	NA	.
rs3860374	chr2:121348224	0.6234	5	NA	.
rs6746992	chr2:121348472	0.6234	5	NA	.
rs6726610	chr2:121350667	0.6085	6	NA	.

Supplementary Table 2 – All SNPs within $r^2 \geq 0.8$ of most associated SNP in the *FLJ14816 | GLI2* region

Coordinates based on GRCh37. r^2 calculated from CEU samples from Phase 3 of the 1000 Genomes Project. RegulomeDB key below.

RegulomeDB Key

1a - eQTL + TF binding + matched TF motif + matched DNase Footprint + DNase peak
1b - eQTL + TF binding + any motif + DNase Footprint + DNase peak
1c - eQTL + TF binding + matched TF motif + DNase peak
1d - eQTL + TF binding + any motif + DNase peak
1e - eQTL + TF binding + matched TF motif
1f - eQTL + TF binding / DNase peak
2a - TF binding + matched TF motif + matched DNase Footprint + DNase peak
2b - TF binding + any motif + DNase Footprint + DNase peak
2c - TF binding + matched TF motif + DNase peak
3a - TF binding + any motif + DNase peak
3b - TF binding + matched TF motif
4 - TF binding + DNase peak
5 - TF binding or DNase peak
6 - other

RS Number	Coordinates	r²	RegulomeDB	Function	Immunochip P-Value
rs9396510	chr6:14560180	Index	5	NA	7.52E-06
rs9396511	chr6:14560204	1	5	NA	.
rs9349947	chr6:14560205	1	5	NA	.
rs9370753	chr6:14560233	1	5	NA	.
rs9396512	chr6:14560320	1	5	NA	.
rs9349948	chr6:14560439	1	4	NA	.
rs9349949	chr6:14560440	1	4	NA	.
rs9396509	chr6:14559900	1	.	NA	.
rs5874476	chr6:14559628	1	.	NA	.
rs1984251	chr6:14559621	1	.	NA	.
rs1984252	chr6:14559575	1	5	NA	.
rs6914991	chr6:14560845	1	2b	NA	.
rs9370754	chr6:14560915	1	5	NA	.
rs1984253	chr6:14559425	1	3a	NA	.
rs9349946	chr6:14559125	1	5	NA	.
rs67605209	chr6:14559001	1	.	NA	.
rs9370752	chr6:14558920	1	6	NA	.
rs9370751	chr6:14558753	1	5	NA	.
rs35738380	chr6:14558346	1	.	NA	.
rs1891283	chr6:14556914	1	6	NA	.
rs9296937	chr6:14555508	1	6	NA	.
rs9396508	chr6:14554970	1	.	NA	.
rs6937863	chr6:14554627	1	.	NA	.
rs9396507	chr6:14554373	1	5	NA	.
rs9396506	chr6:14554009	1	6	NA	.
rs9396505	chr6:14551974	1	5	NA	.
rs9370750	chr6:14551481	1	5	NA	.
rs1001539	chr6:14550793	1	3a	NA	.
rs16875127	chr6:14549785	1	4	NA	.
rs9358037	chr6:14548821	1	.	NA	.
rs2179092	chr6:14547906	1	5	NA	.
rs4331989	chr6:14547702	1	5	NA	.
rs78895936	chr6:14546968	1	6	NA	.
rs58399222	chr6:14546112	1	.	NA	.
rs733792	chr6:14544219	1	5	NA	.
rs7758992	chr6:14541957	1	.	NA	.
rs3817761	chr6:14540994	1	5	NA	.
rs3817760	chr6:14540915	1	5	NA	.
rs3817758	chr6:14540747	1	4	NA	.
rs3817757	chr6:14540738	1	4	NA	.
rs2142608	chr6:14540495	1	5	NA	.
rs9349945	chr6:14539891	1	6	NA	.
rs7774975	chr6:14539376	1	5	NA	.
rs7774347	chr6:14539036	1	5	NA	.
rs9382981	chr6:14561986	0.8704	.	NA	.
rs9349950	chr6:14562744	0.8704	6	NA	.
rs9367843	chr6:14562909	0.8704	6	NA	.
rs75171351	chr6:14563461	0.8704	.	NA	.
rs76743380	chr6:14563586	0.8704	4	NA	.
rs79137793	chr6:14563785	0.8704	4	NA	.

rs6906503	chr6:14564186	0.8704	6	NA	.
rs6901847	chr6:14564196	0.8704	.	NA	.
rs6922613	chr6:14564246	0.8704	.	NA	.
rs6922934	chr6:14564362	0.8704	.	NA	.
rs6922038	chr6:14564648	0.8704	6	NA	.
rs57653370	chr6:14564854	0.8704	6	NA	.
rs9396513	chr6:14565123	0.8704	5	NA	.
rs9396514	chr6:14565438	0.8704	.	NA	.
rs77677669	chr6:14565727	0.8704	.	NA	.
rs9396515	chr6:14565902	0.8704	.	NA	.
rs9370756	chr6:14566072	0.8704	.	NA	.
rs9396516	chr6:14566086	0.8704	6	NA	.
rs9396517	chr6:14566273	0.8704	.	NA	.
rs9370757	chr6:14566428	0.8704	.	NA	.
rs9396518	chr6:14566548	0.8704	.	NA	.
rs9396519	chr6:14566787	0.8704	.	NA	.
rs6919797	chr6:14567096	0.8704	6	NA	.
rs6924439	chr6:14567145	0.8704	6	NA	.
rs9358038	chr6:14568186	0.8704	3a	NA	.
rs9367844	chr6:14568650	0.8704	.	NA	.
rs9296935	chr6:14551477	0.8704	5	NA	.
rs112194433	chr6:14571609	0.8704	6	NA	.
rs6938103	chr6:14572336	0.8704	5	NA	.
rs6939631	chr6:14572428	0.8704	6	NA	.
rs57574258	chr6:14573098	0.8704	6	NA	.
rs74647227	chr6:14573212	0.8704	6	NA	.
rs6459362	chr6:14573435	0.8704	6	NA	.
rs16875212	chr6:14573948	0.8704	5	NA	.
rs9382982	chr6:14574161	0.8704	5	NA	.
rs71782543	chr6:14574318	0.8704	.	NA	.
rs9367845	chr6:14579640	0.8704	.	NA	.

Supplementary Table 3 – All SNPs within r^2≥0.8 of most associated SNP in the *LOC100128517 | LOC728961* region

Coordinates based on GRCh37. r² calculated from CEU samples from Phase 3 of the 1000 Genomes Project. RegulomeDB key below.

RegulomeDB Key

1a - eQTL + TF binding + matched TF motif + matched DNase Footprint + DNase peak
1b - eQTL + TF binding + any motif + DNase Footprint + DNase peak
1c - eQTL + TF binding + matched TF motif + DNase peak
1d - eQTL + TF binding + any motif + DNase peak
1e - eQTL + TF binding + matched TF motif
1f - eQTL + TF binding / DNase peak
2a - TF binding + matched TF motif + matched DNase Footprint + DNase peak
2b - TF binding + any motif + DNase Footprint + DNase peak
2c - TF binding + matched TF motif + DNase peak
3a - TF binding + any motif + DNase peak
3b - TF binding + matched TF motif
4 - TF binding + DNase peak
5 - TF binding or DNase peak
6 - other

Gene	Allele (HLA)	P-Value
HLA_DRB1	HLA_DRB1_0301	5.77E-34
HLA_DRB1	HLA_DRB1_03	7.97E-34
HLA_DQB1	HLA_DQB1_0201	3.88E-33
HLA_B	HLA_B_0801	2.28E-26
HLA_B	HLA_B_08	2.29E-26
HLA_DQA1	HLA_DQA1_05	2.25E-21
HLA_DQA1	HLA_DQA1_0501	2.25E-21
HLA_C	HLA_C_0701	4.93E-20
HLA_DQB1	HLA_DQB1_02	1.66E-17
HLA_DQB1	HLA_DQB1_03	1.09E-14
HLA_DRB1	HLA_DRB1_0101	7.25E-12
HLA_DQA1	HLA_DQA1_0301	8.62E-12
HLA_DQA1	HLA_DQA1_03	8.63E-12
HLA_DRB1	HLA_DRB1_04	5.15E-11
HLA_DRB1	HLA_DRB1_1501	1.61E-09
HLA_A	HLA_A_0101	2.15E-09
HLA_A	HLA_A_01	2.16E-09
HLA_DQA1	HLA_DQA1_0102	4.12E-09
HLA_DQB1	HLA_DQB1_0602	4.54E-09
HLA_DRB1	HLA_DRB1_15	7.15E-09
HLA_DQB1	HLA_DQB1_0501	2.69E-08
HLA_DRB1	HLA_DRB1_0701	3.71E-08
HLA_DRB1	HLA_DRB1_07	3.72E-08
HLA_DRB1	HLA_DRB1_01	4.58E-08
HLA_DQA1	HLA_DQA1_0201	4.59E-08
HLA_DQA1	HLA_DQA1_02	4.60E-08
HLA_DRB1	HLA_DRB1_0401	1.20E-07
HLA_DQB1	HLA_DQB1_0302	1.25E-07
HLA_B	HLA_B_07	6.20E-07
HLA_B	HLA_B_0702	9.43E-07
HLA_DPB1	HLA_DPB1_01	4.85E-06
HLA_DPB1	HLA_DPB1_0101	4.85E-06

Supplementary Table 4 - HLA Imputation in 252 IBM patients

Classical HLA alleles reaching significance ($6.8x10^{-6}$)

Gene	Allele (HLA)	P-Value
HLA_DRB1	HLA_DRB1_0101	1.56E-16
HLA_DQB1	HLA_DQB1_0501	1.04E-13
HLA_DRB1	HLA_DRB1_01	6.34E-13
HLA_DQB1	HLA_DQB1_05	7.10E-11
HLA_DQA1	HLA_DQA1_0101	8.80E-10
HLA_DRB1	HLA_DRB1_1501	1.29E-07
HLA_DRB1	HLA_DRB1_04	2.30E-07
HLA_DQA1	HLA_DQA1_0301	2.52E-07
HLA_DQA1	HLA_DQA1_03	2.52E-07
HLA_DQB1	HLA_DQB1_0602	4.78E-07
HLA_B	HLA_B_3501	1.33E-06
HLA_DQA1	HLA_DQA1_01	2.41E-06
HLA_DQB1	HLA_DQB1_0302	2.78E-06
HLA_DRB1	HLA_DRB1_15	2.92E-06
HLA_DQA1	HLA_DQA1_0201	4.05E-06
HLA_DQA1	HLA_DQA1_02	4.06E-06
HLA_DRB1	HLA_DRB1_0701	4.09E-06
HLA_DRB1	HLA_DRB1_07	4.09E-06

Supplementary Table 5 - HLA Imputation in 252 IBM patients conditional on HLA-DRB1*03:01

Classical HLA alleles reaching significance ($6.8x10^{-6}$)

Gene	Allele (HLA)	P-Value
HLA_DQA1	HLA_DQA1_0103	4.14E-09
HLA_DQB1	HLA_DQB1_0603	2.01E-08
HLA_DRB1	HLA_DRB1_1301	3.28E-08

Supplementary Table 6 - HLA Imputation in 252 IBM patients conditional on HLA-DRB1*03:01 and HLA-DRB1*01:01

Classical HLA alleles reaching significance ($6.8x10^{-6}$)

Gene	Amino acid (AA)	P-Value
DRB1	AA_DRB1_26	5.22E-43
DRB1	AA_DRB1_74	3.31E-41
DRB1	AA_DRB1_11	1.88E-37
DRB1	AA_DRB1_13	3.00E-37
DQA1	AA_DQA1_53	4.52E-35
DQA1	AA_DQA1_50	4.52E-35
DQA1	AA_DQA1_47	4.40E-34
DRB1	AA_DRB1_77	7.69E-34
DRB1	AA_DRB1_37	1.07E-32
DQA1	AA_DQA1_175	3.19E-31
DRB1	AA_DRB1_104	2.81E-29
DRB1	AA_DRB1_98	2.81E-29
DQB1	AA_DQB1_30	2.91E-29
DQA1	AA_DQA1_215	3.40E-29
DQB1	AA_DQB1_74	5.96E-29
DQB1	AA_DQB1_71	5.39E-28
B	AA_B_67	1.91E-27
B	AA_B_9	2.34E-27
DQB1	AA_DQB1_75	2.24E-25
DQB1	AA_DQB1_38	5.00E-24
DRB1	AA_DRB1_96	9.03E-24
DQB1	AA_DQB1_55	1.41E-23
DQB1	AA_DQB1_77	1.94E-23
DQA1	AA_DQA1_107	6.75E-23
DQA1	AA_DQA1_161	7.36E-23
DQA1	AA_DQA1_163	7.36E-23
DQA1	AA_DQA1_156	7.41E-23
DQA1	AA_DQA1_75	2.25E-21
DQA1	AA_DQA1_51	2.33E-21
DQA1	AA_DQA1_40	2.33E-21
B	AA_B_163	9.92E-21
DRB1	AA_DRB1_67	4.10E-20
DRB1	AA_DRB1_32	6.75E-20
C	AA_C_66	8.33E-20
DRB1	AA_DRB1_149	2.30E-19
DRB1	AA_DRB1_10	3.38E-19
DRB1	AA_DRB1_-25	4.58E-19
DRB1	AA_DRB1_-16	4.67E-19
DRB1	AA_DRB1_233	4.67E-19
DQB1	AA_DQB1_67	9.01E-19
DQB1	AA_DQB1_66	9.10E-19
DRB1	AA_DRB1_71	2.39E-18
DQB1	AA_DQB1_37	3.20E-18
DRB1	AA_DRB1_12	1.01E-17
DQB1	AA_DQB1_-10	1.01E-17
DQB1	AA_DQB1_28	1.65E-17
DQB1	AA_DQB1_52	1.67E-17
DQB1	AA_DQB1_46	1.68E-17
DQB1	AA_DQB1_47	1.68E-17
DQA1	AA_DQA1_56	2.75E-17
DQA1	AA_DQA1_76	2.75E-17

DQA1	AA_DQA1_187	3.18E-16
B	AA_B_156	4.41E-16
DRB1	AA_DRB1_60	4.99E-16
DRB1	AA_DRB1_57	2.94E-15
DRB1	AA_DRB1_30	9.11E-15
DRB1	AA_DRB1_180	1.56E-14
DRB1	AA_DRB1_120	1.67E-14
DRB1	AA_DRB1_-24	2.51E-14
DQB1	AA_DQB1_140	3.13E-14
DQB1	AA_DQB1_182	3.17E-14
DRB1	AA_DRB1_73	2.75E-12
DQB1	AA_DQB1_57	2.86E-12
B	AA_B_178	3.77E-12
DRB1	AA_DRB1_4	3.81E-12
DQA1	AA_DQA1_26	8.62E-12
DQA1	AA_DQA1_52	2.70E-11
DRB1	AA_DRB1_33	5.07E-11
DQA1	AA_DQA1_207	6.52E-11
DQB1	AA_DQB1_185	9.03E-11
DRB1	AA_DRB1_-1	1.14E-10
DRB1	AA_DRB1_181	1.70E-10
DQA1	AA_DQA1_34	4.15E-10
B	AA_B_63	2.00E-09
A	AA_A_150	2.15E-09
A	AA_A_158	2.16E-09
A	AA_A_44	2.60E-09
A	AA_A_67	2.80E-09
A	AA_A_163	3.14E-09
B	AA_B_24	3.26E-09
A	AA_A_90	7.89E-09
DRB1	AA_DRB1_47	8.21E-09
DRB1	AA_DRB1_78	9.65E-09
A	AA_A_152	1.03E-08
B	AA_B_41	1.24E-08
B	AA_B_69	1.26E-08
B	AA_B_45	1.56E-08
DRB1	AA_DRB1_142	1.64E-08
DRB1	AA_DRB1_133	1.67E-08
DRB1	AA_DRB1_25	3.66E-08
DRB1	AA_DRB1_14	3.71E-08
DRB1	AA_DRB1_70	3.82E-08
DQA1	AA_DQA1_54	4.59E-08
A	AA_A_156	5.71E-08
B	AA_B_32	7.04E-08
B	AA_B_12	8.42E-08
B	AA_B_113	9.81E-08
B	AA_B_70	1.08E-07
B	AA_B_71	1.13E-07
DQB1	AA_DQB1_9	1.94E-07
DQB1	AA_DQB1_135	2.73E-07
B	AA_B_114	4.68E-07
B	AA_B_97	8.09E-07

A	AA_A_76	1.02E-06
DQB1	AA_DQB1_-18	1.10E-06
DQB1	AA_DQB1_125	1.18E-06
A	AA_A_105	1.18E-06
A	AA_A_276	1.78E-06
A	AA_A_321	1.83E-06
DRB1	AA_DRB1_31	2.01E-06
DQB1	AA_DQB1_26	2.14E-06
DQB1	AA_DQB1_-5	2.45E-06
B	AA_B_11	2.51E-06
C	AA_C_99	4.88E-06
DPB1	AA_DPB1_194	4.95E-06
A	AA_A_97	5.64E-06

Supplementary Table 7 - HLA amino acid imputation in 252 IBM patients

Amino acids reaching significance ($6.8x10^{--6}$)

Gene	Amino acid (AA)	P-Value
DRB1	AA_DRB1_11	3.80E-13
DRB1	AA_DRB1_104	3.69E-11
DRB1	AA_DRB1_98	3.69E-11
DQA1	AA_DQA1_215	4.08E-11
DQA1	AA_DQA1_53	1.52E-10
DQA1	AA_DQA1_50	1.52E-10
DRB1	AA_DRB1_149	3.06E-10
DRB1	AA_DRB1_10	4.22E-10
DRB1	AA_DRB1_12	4.22E-10
DRB1	AA_DRB1_13	5.24E-10
DQA1	AA_DQA1_47	8.32E-10
DRB1	AA_DRB1_233	8.91E-10
DRB1	AA_DRB1_.25	2.54E-09
DRB1	AA_DRB1_.16	2.56E-09
DRB1	AA_DRB1_57	3.02E-09
DQA1	AA_DQA1_41	3.14E-09
DQA1	AA_DQA1_130	3.37E-09
DRB1	AA_DRB1_60	3.54E-09
DRB1	AA_DRB1_37	7.84E-09
DRB1	AA_DRB1_74	6.08E-08
DRB1	AA_DRB1_181	1.80E-07
DQA1	AA_DQA1_34	3.19E-07
DRB1	AA_DRB1_30	3.99E-07
B	AA_B_67	4.75E-07
DQA1	AA_DQA1_26	7.81E-07
DQA1	AA_DQA1_187	8.14E-07
DQB1	AA_DQB1_30	1.06E-06
DRB1	AA_DRB1_4	1.44E-06
DRB1	AA_DRB1_78	1.49E-06
DRB1	AA_DRB1_28	2.23E-06
DQA1	AA_DQA1_56	4.15E-06
DQA1	AA_DQA1_76	4.15E-06
DRB1	AA_DRB1_70	4.16E-06
DRB1	AA_DRB1_96	5.46E-06
DQA1	AA_DQA1_175	6.31E-06

Supplementary Table 8 - HLA amino acid imputation in 252 IBM patients conditional on HLA-DRB1 amino acid position 26

Amino acids reaching significance ($6.8x10^{-6}$)

Gene	Allele (HLA) / Amino acid (AA)	P-Value
DQA1	AA_DQA1_53	5.49E-13
DQA1	AA_DQA1_50	5.49E-13
DRB1	AA_DRB1_13	5.93E-13
DQA1	AA_DQA1_47	1.62E-12
DRB1	AA_DRB1_11	1.81E-12
DRB1	AA_DRB1_74	1.95E-11
DQA1	AA_DQA1_215	8.38E-11
DRB1	AA_DRB1_104	8.40E-11
DRB1	AA_DRB1_98	8.40E-11
DQA1	AA_DQA1_175	1.46E-10
DRB1	AA_DRB1_26	2.67E-09
DRB1	AA_DRB1_149	6.65E-09
DRB1	AA_DRB1_10	7.36E-09
DQA1	AA_DQA1_107	3.98E-08
DQA1	AA_DQA1_156	3.98E-08
DQA1	AA_DQA1_161	3.98E-08
DQA1	AA_DQA1_163	3.98E-08
HLA_DRB1	HLA_DRB1_03	5.58E-08
DRB1	AA_DRB1_77	5.61E-08
HLA_DRB1	HLA_DRB1_0301	5.62E-08
DRB1	AA_DRB1_-16	6.18E-08
DRB1	AA_DRB1_-25	6.18E-08
HLA_DQB1	HLA_DQB1_0201	1.72E-07
DRB1	AA_DRB1_233	2.20E-07
DRB1	AA_DRB1_96	3.42E-07
DRB1	AA_DRB1_12	6.16E-07
DQA1	AA_DQA1_40	7.03E-07
DQA1	AA_DQA1_51	7.03E-07
DQA1	AA_DQA1_75	8.41E-07
HLA_DQA1	HLA_DQA1_05	8.41E-07
HLA_DQA1	HLA_DQA1_0501	8.41E-07
DRB1	AA_DRB1_37	1.96E-06
DQA1	AA_DQA1_187	2.90E-06
DQA1	AA_DQA1_56	3.42E-06
DQA1	AA_DQA1_76	3.42E-06
DRB1	AA_DRB1_120	5.94E-06

Supplementary Table 9 - HLA Imputation in 35 anti-CN-1A positive patients and 140 healthy controls

HLA alleles and amino acids reaching significance ($6.8x10^{-6}$)

HLA allele or Genotype	Number of individuals (present/absent)	Mean age of onset present (years)	Mean age of onset absent (years)	p-value
**DRB1*01:01**	48/76	61.5	60	0.44
**DRB1*03:01**	92/32	60.5	60.8	0.86
**DRB1*13:01**	24/100	61.7	60.3	0.52
**DRB1*01:01/01:01**	4/120	68	60.3	0.1
**DRB1*03:01/03:01**	5/119	56.3	60.8	0.31
**DRB1*13:01/13:01**	0/124	N/A	N/A	N/A
**DRB1*01:01/03:01**	35/89	60.7	60.5	0.92
**DRB1*03:01/13:01**	15/109	63.1	60.2	0.3
**DRB1*01:01/13:01**	3/121	62.6	60.5	0.72
**DRB1*01:01/Non-risk**	6/118	60.8	60.6	0.95
**DRB1*03:01/Non-risk**	37/87	59.8	60.9	0.56
**DRB1*13:01/Non-risk**	6/118	58	60.7	0.51
**Non-risk/Non-risk**	13/111	59.6	60.7	0.71

Supplementary Table 10 - Linear regression analysis for the relationship of HLA-DRB1 genotypes and age of onset in 124 IBM patients

*non-risk defined as any allele not previously reported as conferring an earlier age of onset (HLA-DRB1*01:01, HLA-DRB1*03:01 and HLA-DRB1*13:01)
