# Supplementary material for: Immune‐Array Analysis in Sporadic Inclusion Body Myositis Reveals HLA–DRB1 Amino Acid Heterogeneity Across the Myositis Spectrum
Source: Arthritis Rheumatol. 2017 Apr 4;69(5):1090–9. doi: 10.1002/art.40045 (PMC5516174; doi:10.1002/art.40045)
Supplement: Supplementary file 2 — Supplementary Materials 2 [file ART-69-1090-s002.doc]

Supplementary Materials

Shared Immunochip Control Cohorts

We thank the Rheumatoid Arthritis Consortium International (RACI) for Netherlands, Spanish,

Swedish, UK and US Immunochip control genotypes. This study makes use of data generated by the

Wellcome Trust Case-Control Consortium. A full list of the investigators who contributed to the

generation of the data is available from www.wtccc.org.uk. Funding for the project was provided by

the Wellcome Trust under award 076113 and 085475. Swedish control data was provided from EIRA

study, Professor Lars Alfredsson, Department of Environmental Medicine, Karolinska Institutet,

Stockholm, Sweden. Control data from the Netherlands was provided from the department of

Rheumatology, Leiden University Medical Center, Leiden, The Netherlands.

Polish control data was provided by the Celiac Disease Consortium and Hungarian control data

collected with the help of the Hungarian Research Fund (OTKA) grant K101788 was provided by Prof

Ilma.Korponay-Szabo, Celiac Disease Centre, Heim Pál Children's Hospital, Budapest and University

of Debrecen, Debrecen Hungary.

We acknowledge the International MS Genetics Consortium for providing access to control sample

data from Belgium, France, Norway, Italy and Germany. The collection and genotyping of these

samples was made possible by: the Norwegian MS society and the Norwegian Bone Marrow Registry

(Norwegian samples); the French Biological Resource Center for MS Genetics, Genethon and INSERM

(French samples); and a FISM (Italian Foundation for Multiple Sclerosis) grant (“Progetto Speciale

Immunochip”) for Italian samples.

Italian samples were collected by Prof. Sandra D'Alfonso (Interdisciplinary Research Center of

Autoimmune Diseases IRCAD, University of Eastern Piedmont, Novara, Italy; PROGEMUS

Consortium) and Dr. Martinelli Boneschi (Laboratory of Genetics of Complex Neurological Disorders,

Division of Neuroscience & INSPE, San Raffaele Scientific Institute, Milan, Italy; PROGRESSO

Consortium); funding was provided by a FISM (Italian Foundation for Multiple Sclerosis) grant

(“Progetto Speciale Immunochip). The KORA study was initiated and financed by the Helmholtz

Zentrum München – German Research Center for Environmental Health, which is funded by the

German Federal Ministry of Education and Research (BMBF) and by the State of Bavaria.

Furthermore, KORA research was supported within the Munich Center of Health Sciences (MCHealth),

Ludwig-Maximilians-Universität, as part of LMUinnovativ.

UK Adult Onset Myositis Immunogenetic Collaboration (UKMYONET)

Members of the UK Adult Onset Myositis Immunogenetic Collaboration who recruited and enrolled

subjects are as follows: Drs. Yasmeen Ahmed (Llandudno General Hospital), Raymond Armstrong

(Southampton General Hospital), Robert Bernstein (Manchester Royal Infirmary), Carol Black (Royal

Free Hospital, London), Simon Bowman (University Hospital, Birmingham), Ian Bruce (Manchester

Royal Infirmary), Robin Butler (Robert Jones & Agnes Hunt Orthopaedic Hospital, Oswestry), John

Carty (Lincoln County Hospital), Chandra Chattopadhyay (Wrightington Hospital), Easwaradhas

Chelliah (Wrightington Hospital), Fiona Clarke (James Cook University Hospital, Middlesborough),

Peter Dawes (Staffordshire Rheumatology Centre, Stoke on Trent), Joseph Devlin (Pinderfields

General Hospital, Wakefield), Christopher Edwards (Southampton General Hospital), Paul Emery

(Academic Unit of Musculoskeletal Disease, Leeds), John Fordham (South Cleveland Hospital,

Middlesborough), Alexander Fraser (Academic Unit of Musculoskeletal Disease, Leeds), Hill Gaston

(Addenbrooke's Hospital, Cambridge), Patrick Gordon (King's College Hospital, London), Bridget

Griffiths (Freeman Hospital, Newcastle), Harsha Gunawardena (Frenchay Hospital, Bristol), Frances

Hall (Addenbrooke's Hospital, Cambridge), Beverley Harrison (North Manchester General Hospital),

Elaine Hay (Staffordshire Rheumatology Centre, Stoke on Trent), Lesley Horden (Dewsbury District

General Hospital), John Isaacs (Freeman Hospital, Newcastle), Adrian Jones (Nottingham University

Hospital), Sanjeet Kamath (Staffordshire Rheumatology Centre, Stoke on Trent), Thomas Kennedy

(Royal Liverpool Hospital), George Kitas (Dudley Group Hospitals Trust, Birmingham), Peter Klimiuk

(Royal Oldham Hospital), Sally Knights (Yeovil District Hospital, Somerset), John Lambert (Doncaster

Royal Infirmary), Peter Lanyon (Queen's Medical Centre, Nottingham), Ramasharan Laxminarayan

(Queen's Hospital, Burton Upon Trent), Bryan Lecky (Walton Neuroscience Centre, Liverpool),

Raashid Luqmani (Nuffield Orthopaedic Centre, Oxford), Jeffrey Marks (Steeping Hill Hospital,

Stockport), Michael Martin (St. James University Hospital, Leeds), Dennis McGonagle (Academic Unit

of Musculoskeletal Disease, Leeds), Neil McHugh (Royal National Hospital for Rheumatic Diseases,

Bath), Francis McKenna (Trafford General Hospital, Manchester), John McLaren (Cameron Hospital,

Fife), Michael McMahon (Dumfries & Galloway Royal Infirmary, Dumfries), Euan McRorie (Western

General Hospital, Edinburgh), Peter Merry (Norfolk & Norwich University Hospital, Norwich), Sarah

Miles (Dewsbury & District General Hospital, Dewsbury), James Miller (Royal Victoria Hospital,

Newcastle), Anne Nicholls (West Suffolk Hospital, Bury St. Edmunds), Jennifer Nixon (Countess of

Chester Hospital, Chester), Voon Ong (Royal Free Hospital, London), Katherine Over (Countess of

Chester Hospital, Chester), John Packham (Staffordshire Rheumatology Centre, Stoke on Trent),

Nicolo Pipitone (King's College Hospital, London), Michael Plant (South Cleveland Hospital,

Middlesborough), Gillian Pountain (Hinchingbrooke Hospital, Huntington), Thomas Pullar (Ninewells

Hospital, Dundee), Mark Roberts (Salford Royal Foundation Trust), Paul Sanders (Wythenshawe

Hospital, Manchester), David Scott (King's College Hospital, London), David Scott (Norfolk & Norwich

University Hospital, Norwich), Michael Shadforth (Staffordshire Rheumatology Centre, Stoke on

Trent), Thomas Sheeran (Cannock Chase Hospital, Cannock, Staffordshire), Arul Srinivasan

(Broomfield Hospital, Chelmsford), David Swinson (Wrightington Hospital), Lee-Suan Teh (Royal

Blackburn Hospital, Blackburn), Michael Webley (Stoke Manderville Hospital, Aylesbury), Brian

Williams (University Hospital of Wales, Cardiff), and Jonathan Winer (Queen Elizabeth Hospital,

Birmingham).
